# Hemoglobin A1c Level Is Not Related to the Severity of Atherosclerosis in Patients with Acute Coronary Syndrome

**DOI:** 10.1155/2015/192108

**Published:** 2015-07-16

**Authors:** Xinhong Wang, Zhenhua Han, Guanghua Hao, Yongqin Li, Xin Dong, Congxia Wang

**Affiliations:** Department of Cardiovascular Medicine, Second Affiliated Hospital of Medical College, Xi'an Jiaotong University, Xi'an, Shaanxi 710004, China

## Abstract

*Background*. The relationship between hemoglobin A1c (HbA1c) levels and the extent of coronary artery stenosis in patients with acute coronary syndrome (ACS) remains uncertain. The present study aimed to assess the correlation of HbA1c level with angiographic coronary atherosclerosis. *Methods*. 292 consecutive ACS patients were enrolled and stratified into three groups according to HbA1c levels (group 1: <6.0%, *n* = 137; group 2: 6.0–6.4%, *n* = 67; group 3: ≥6.5%, *n* = 88). The severity of coronary arteriosclerosis was assessed by Gensini score. The relationship between HbA1c and Gensini score was analyzed by multiple variables analysis. *Results*. HbA1c level was not associated with the severity of CAD assessed by Gensini score in patients with ACS, even after the adjustment for other risk factors. However, NT-proBNP, ApoA1 and LVEF levels were independent predictors for CAD severity. Moreover, HbA1c level was not associated with the risk of high Gensini score (>40) by logistic regression analysis. Diabetes mellitus (DM) and LVEF levels were two independent risk factors for high Gensini score. *Conclusions*. HbA1c level is not a significant and independent marker for the severity of angiography in ACS patients, even in high-risk patients.

## 1. Introduction

Acute coronary syndrome (ACS) indicates serious clinical manifestation of coronary artery disease (CAD) and is the major cause of morbidity and mortality worldwide. The severity of coronary atherosclerosis is closely associated with cardiovascular prognosis in patients with ACS [1]. Consequently, prediction and diagnosis of the extent of coronary lesion in ACS are important for clinical management of this disease.

Haemoglobin (HbA1c) concentration is an established marker of average blood glucose concentration and has been suggested as a diagnostic or screening tool for diabetes [2]. Elevated HbA1c levels in patients with or without diabetes mellitus (DM) are associated with an increased risk for cardiovascular disease and mortality [3, 4]. A community-based population study including 11,092 patients without DM found that elevated HbA1c level was strongly associated with the risks of cardiovascular disease and mortality [4]. Timmer et al. demonstrated that elevated HbA1c level was associated with adverse outcome in nondiabetic patients with ST-segment-elevation myocardial infarction (STEMI) [5]. On the other hand, an increase of 1% in HbA1c concentration was associated with roughly a 30% increase in all cause and 40% increase in cardiovascular or ischaemic heart disease mortality among individuals with diabetes [6]. However, the prognostic role of HbA1c in the acute phase in patients with STEMI is not clear [7]. A recent study reported that there was no overall association between HbA1c level and the prognosis of nondiabetic patients with suspected stable angina pectoris (SAP) [8]. However, another study found that HbA1c appeared to be an independent predictor for the severity of CAD and poor outcome in patients with stable angina [9].

To the best of our knowledge, the relationship between HbA1c level and the extent of coronary artery stenosis in patients with ACS remains uncertain. Therefore, this study aimed to assess the relationship of HbA1c level with the extent of angiographic coronary atherosclerosis.

## 2. Methods

### 2.1. Patients

The study recruited 292 consecutive ACS patients admitted to Cardiology Department of the Second Affiliated Hospital of Medical College of Xi'an Jiaotong University from January 2012 to July 2014. All patients, including unstable angina, non-ST-segment-elevation myocardial infarction, and ST-segment-elevation MI, met the ACS diagnostic criteria outlined in the ACC/AHA 2007 guidelines. Exclusion criteria included advanced liver or renal dysfunction, coronary bypass surgery, cancer, active or chronic inflammatory or autoimmune diseases, acute infection, and hematological diseases. Informed written consent was obtained from all patients, and the study complied with the Declaration of Helsinki and was approved by the Ethics Committee of our hospital. Cardiovascular risk factors and cardiovascular medications were recorded by standardized questionnaire. The left ventricular ejection fraction (LVEF) was evaluated by echocardiograph.

### 2.2. Laboratory Tests

Peripheral blood samples were drawn routinely from patients in a fasting state. Plasma concentrations of glucose, lipids, lipoproteins, and other biochemical parameters were determined at the Biochemistry Department in our hospital. High-sensitivity C-reactive protein (hsCRP) level was analyzed by a highly sensitive ABC-double-antibody sandwich ELISA (R&D). HbA1c level was determined using cation exchange column chromatography on an automated HPLC instrument (Variant II Turbo, Bio-Rad Laboratories, Hercules, California, USA). NT-proBNP was measured using electrochemiluminescence-based immunoanalytical system (Roche Diagnostics Ltd., Mannheim, Germany).

### 2.3. Coronary Angiography

Coronary angiography was analyzed by two independent experienced cardiologists. CAD was defined as any main branch with coronary artery stenosis ≥50%. The burden of coronary arteriosclerosis was assessed by Gensini score system [10]. Reductions in lumen diameter of 25%, 50%, 75%, 90%, 99%, and complete occlusion were evaluated as 1, 2, 4, 8, 16, and 32, respectively. The stenosis score was multiplied by a factor taking into account the position of the coronary lesions: 5 for the left main coronary artery, 2.5 for the proximal left anterior descending artery or proximal left circumflex artery, 1.5 for the mid-region of the left anterior descending artery, 1 for the distal left anterior descending artery, the mid-distal region of the left circumflex artery or right coronary artery, and 0.5 for other segments.

### 2.4. Statistical Analysis

All statistical analyses were performed using the SPSS software package version 18.0 (SPSS Inc., Chicago, IL, USA). Quantitative variables were expressed as means ± standard deviation and categorical variables as frequency and percentage. The Shapiro-Wilk test was performed to assess normal distribution of quantitative variables. Nonnormally distributed variables such as NT-proBNP and hsCRP were log-transformed. Analysis of variance (ANOVA), *t*-test, or chi-square test was used for the comparisons of the groups. A multiple linear regression model was used to assess the association between HbA1c and Gensini score. A binary logistic regression model using stepwise selection process for the prediction of high-risk coronary lesions (Gensini score > 40) was calculated. A 2-tailed *P* < 0.05 was considered statistically significant.

## 3. Results

### 3.1. Baseline Characteristics of Patients

The demographic and clinical characteristics of patients in different groups according to HbA1c levels were shown in Table 1. No difference in Gensini score was found in different groups (*P* = 0.085). Intergroup comparisons showed that patients with higher HbA1c level had higher fasting blood glucose and hsCRP levels. The frequency distribution of older subjects differed significantly in three groups. There were no significant differences in other characteristics among the three groups.

### 3.2. Association of HbA1c Level with Coronary Atherosclerosis

**Figure 1:**
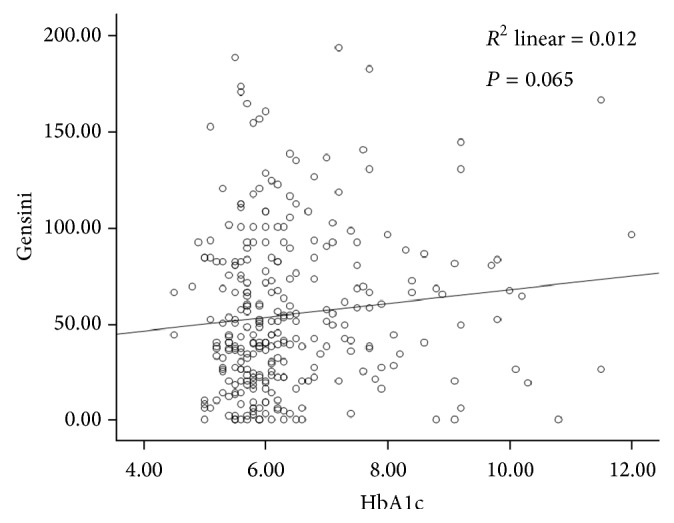
Correlation between HbA1c level and Gensini score in patients with ACS.

To explore the association of the extent of coronary angiographic results with HbA1c level, a multiple linear regression analysis using stepwise selection process was performed to adjust for the variables which are likely to be independently associated with Gensini score. Age, gender, smoking, ventricular function, NT-proBNP, ApoA1, heart rate, HDL-C, fasting blood glucose, and hsCRP were adjusted. No correlation was found between HbA1c level and the extent of coronary atherosclerosis in ACS, as determined with the Gensini score (*β* = 0.079, *P* = 0.224). Both fasting blood glucose and hsCRP levels showed no association with the Gensini score. The relationship between HbA1c level and the Gensini score in patients with ACS was shown in Figure 1. However, we found that NT-proBNP, LVEF, ApoA1, and HDL-C levels were independent predictors of Gensini score (Table 2).

### 3.3. Correlation between HbA1c Level and High Gensini Score

To further explore the association of HbA1c level with high Gensini score (>40), a binary logistic regression analysis using stepwise selection process was applied for determining the predictors of high Gensini score. Variables in this model included the age, gender, smoking, diabetes, ventricular function, NT-proBNP, ApoA1, HDL-C, and fasting blood glucose. Notably, diabetic patients included those with previous history of diabetes and those with undiagnosed diabetes according to the new HbA1c cut-off points for diagnosis of diabetes [11]. Patients with HbA1c level ≥6.5% were diagnosed as diabetic, even without previous history of diabetes. HbA1c level was not associated with the risk of high Gensini score. This model demonstrated that diabetes and LVEF levels were two independent risk factors for high Gensini score: odds ratio (OR): 1.982, 95% confidence interval (CI): 1.09, 3.59, *P* < 0.05; OR: 0.933, 95% CI: 0.91, 0.96, *P* < 0.001, respectively (Table 3).

## 4. Discussion

The present study aimed to evaluate the association of HbA1c level and the severity and extent of atherosclerotic lesion of coronary arteries in Chinese patients with ACS. The main finding of our study is that HbA1c level was not associated with the severity of CAD assessed by Gensini score in patients with ACS, even after adjustment for other related factors. However, we demonstrated that NT-proBNP, ApoA1, and LVEF levels were significant independent predictors for CAD severity.

HbA1c has been used to monitor long-term glycemic control in diabetic patients and to diagnose DM [12]. Elevated HbA1c level was associated with the morbidity and mortality of cardiovascular disease in patients with or without DM, even when fasting glucose values were normal [3, 4]. However, the prognostic value of HbA1c in patients with ACS remains controversial. Lazzeri et al. reported that HbA1c was not associated with the mortality in STEMI patients with known diabetes submitted to mechanical revascularization [13]. Cicek et al. showed that HbA1c was an independent predictor of the in-hospital mortality in STEMI patients treated with PCI after adjusting the baseline characteristics [14]. Timmer et al. found that elevated HbA1c level was associated with increased mortality rates over an average 3.3 years of follow-up in 4176 nondiabetic patients with STEMI submitted to PCI [5]. Liu et al. conducted a systematic review on patients hospitalized with CAD and did not detect a relationship between HbA1c level and mortality risk in diabetes [15].

The severity of coronary atherosclerosis is related to cardiovascular outcomes. Gensini score effectively reflects the severity of coronary stenoses and thus predicts the risk of cardiovascular events [1, 16]. A combination of cardiovascular risk factors and Gensini score may provide valuable information on cardiovascular prognosis. However, our study revealed no significant correlation between HbA1c level and Gensini score by using a multiple linear regression model, suggesting that HbA1c level is not a major predictor for the extent of coronary atherosclerosis. Moreover, hsCRP level showed no significant association with CAD severity in the present study although it has been proposed as a prognostic factor for predicting major cardiovascular events and mortality [17]. Our results are consistent with a previous study by Ertem et al. who showed no significant relationship between HbA1c level and Gensini score in patients with ACS without DM [18]. To the best of our knowledge, no study has reported the relationship between HbA1c level and the severity of CAD evaluated with Gensini score in ACS patients with or without DM.

Several potential limitations of our study should be noted. First, the subjects in our study are exclusively limited to Chinese patients, so that conclusions should be drawn cautiously to other ethnic groups. Second, this retrospective study has a relatively small sample size from a single center. Therefore, it will be more powerful if a larger sample size is employed. Finally, intravascular ultrasonography (IVUS) may provide accurate information on atherosclerotic lesions, but no subjects in our study were evaluated by IVUS.

In conclusion, our data suggest that HbA1c level is not a significant and independent maker for the severity of angiography in ACS patients, even if in high-risk patients.

## Figures and Tables

**Table 1:** Baseline clinical characteristics according to HbA1c level.

Variables		HbA1c		*P*
	1 (<6.0%)	2 (6.0~6.4%)	3 (≥6.5%)	
Age (years)	57.19 ± 12.33	62.66 ± 10.17	62.08 ± 12.21	<0.001
Male, *n* (%)	106 (0.77)	54 (0.81)	59 (0.67)	0.110
Hypertension, *n* (%)	63 (0.46)	33 (0.49)	31 (0.35)	0.160
DM, *n* (%)	6 (0.04)	7 (0.10)	61 (0.71)	<0.001
Smoking, *n* (%)	77 (0.56)	43 (0.64)	41 (0.47)	0.090
Previous MI, *n* (%)	15 (0.11)	7 (0.10)	6 (0.07)	0.570
Previous PCI, *n* (%)	11 (0.08)	7 (0.10)	7 (0.08)	0.821
SBP (mmHg)	128.23 ± 22.98	125.39 ± 25.89	129.45 ± 21.76	0.551
DBP (mmHg)	79.32 ± 14.54	78.91 ± 16.18	79.23 ± 11.63	0.981
HR (bpm)	74.74 ± 13.37	73.94 ± 14.52	74.03 ± 15.35	0.905
LVEF (%)	60.67 ± 11.00	58.29 ± 13.65	57.09 ± 12.41	0.095
TC (mmol/L)	3.83 ± 0.98	3.96 ± 0.99	3.80 ± 0.90	0.566
TG (mmol/L)	1.66 ± 1.08	1.54 ± 0.83	1.81 ± 1.15	0.283
HDL-C (mmol/L)	0.92 ± 0.21	0.95 ± 0.24	0.87 ± 0.21	0.080
LDL-C (mmol/L)	2.29 ± 0.87	2.37 ± 0.93	2.26 ± 0.75	0.709
ApoA1 (g/L)	1.07 ± 0.17	1.09 ± 0.19	1.06 ± 0.19	0.558
ApoB (g/L)	0.76 ± 0.23	0.79 ± 0.22	0.78 ± 0.20	0.634
Lp(a) (mg/L)	189.87 ± 141.33	268.17 ± 256.97	200.46 ± 162.24	<0.05
FBS (mmol/L)	6.03 ± 1.97	6.33 ± 1.78	9.81 ± 4.78	<0.001
NT-proBNP (pg/mL)	863.13 ± 2648.72	1546.72 ± 3747.96	1621.95 ± 3029.24	0.153
hsCRP (mg/L)	3.56 ± 3.58	4.24 ± 3.93	5.30 ± 4.27	<0.01
ACEI/ARB, *n* (%)	117 (0.86)	62 (0.94)	74 (0.85)	0.196
*β*-blocker, *n* (%)	122 (0.90)	56 (0.85)	77 (0.89)	0.60
Statins, *n* (%)	136 (1.00)	64 (0.97)	84 (0.97)	0.102
Gensini score	49.66 ± 41.10	54.28 ± 38.82	62.19 ± 42.96	0.085

HbA1c, glycosylatedhemoglobinA1c; DM, diabetes mellitus; MI, myocardial infarction; PCI, percutaneous coronary intervention; SBP, systolic blood pressure; DBP, diastolic blood pressure; HR, heart rate; LVEF, left ventricular ejection fraction; TC, total cholesterol; TG, triglycerides; HDL-C, high-density lipoprotein cholesterol; LDL-C, low-density lipoprotein cholesterol; ApoA1, apolipoprotein A1; ApoB, apolipoprotein B; Lp(a), lipoprotein (a); FBS, fasting blood sugar; hsCRP, high-sensitivity C-reactive protein; ACEI, angiotensin converting enzyme inhibition; ARB, angiotensin receptor blocker.

**Table 2:** Predictors of Gensini score: multivariate linear regression analysis.

	Coefficient	95% CI	*P*
logNT-proBNP	11.24	1.86, 20.63	<0.05
ApoA1	−79.78	−135.39, −24.16	<0.01
LVEF	−0.56	−1.11, −0.01	<0.05
HDL-C	46.07	0.15, 91.98	<0.05

**Table 3:** Predictors of high Gensini score: multivariate logistic regression analysis.

Variables	Coefficient	OR	95% CI	*P*
LVEF	−0.069	0.933	0.91, 0.96	<0.001
DM	0.684	1.982	1.09, 3.59	<0.05
